# Body Mass Index-Related Alterations in Gonadotropin Secretion Among Women with Polycystic Ovary Syndrome Compared with Healthy Controls

**DOI:** 10.3390/diseases14090341

**Published:** 2026-09-16

**Authors:** Saad Al-Fawaeir

**Affiliations:** Department of Medical Laboratory Sciences, Faculty of Allied Medical Sciences, Jadara University, Irbid 21110, Jordan; s.alfawaeir@jadar.edu.jo

**Keywords:** hormonal assessment, obesity, polycystic ovary syndrome, body mass index

## Abstract

Aim: To investigate the association between body mass index (BMI) and gonadotropin secretion, including serum luteinizing hormone (LH), follicle-stimulating hormone (FSH), and the LH/FSH ratio, among women with polycystic ovary syndrome (PCOS), and to compare these parameters with healthy women. Patients and Methods: This comparative cross-sectional analytical study included 400 women aged 18–35 years recruited from outpatient gynecology and endocrinology clinics at private-sector medical centers in Irbid, Jordan, between June 2025 and April 2026. The study included 200 women diagnosed with PCOS according to the Rotterdam criteria and 200 age-comparable healthy controls. The PCOS group was stratified into four BMI categories (<25, 25–<28, 28–<31, and ≥31 kg/m^2^), with 50 women in each category. Clinical and anthropometric characteristics were assessed, and serum LH and FSH concentrations were measured. The LH/FSH ratio was calculated for each participant. Differences between groups were evaluated using appropriate statistical tests, while one-way analysis of variance was used to compare PCOS subgroups according to BMI. Pearson’s correlation analysis was used to assess associations between BMI and gonadotropin parameters. Results: The mean age was comparable between women with PCOS and healthy controls (26.31 ± 5.36 vs. 26.47 ± 5.16 years; *p* = 0.23). Women with PCOS had significantly higher BMI than controls (27.85 ± 3.47 vs. 22.81 ± 2.29 kg/m^2^; *p* < 0.001). Among women with PCOS, menstrual irregularity, hirsutism, acne, infertility, and polycystic ovarian morphology were observed in 87.5%, 79.5%, 78.5%, 70.0%, and 100%, respectively. Serum LH was significantly higher in women with PCOS than in controls (8.44 ± 2.61 vs. 6.53 ± 1.16 IU/L; *p* < 0.001), whereas FSH was significantly lower (4.64 ± 0.98 vs. 7.96 ± 1.18 IU/L; *p* < 0.001), resulting in a higher LH/FSH ratio (1.84 ± 0.50 vs. 0.83 ± 0.20; *p* < 0.001). Within the PCOS group, LH progressively decreased across increasing BMI categories, from 11.58 ± 1.26 IU/L in women with BMI < 25 kg/m^2^ to 5.40 ± 1.29 IU/L in those with BMI ≥ 31 kg/m^2^ (*p* < 0.001). Similarly, the LH/FSH ratio decreased from 2.46 ± 0.18 to 1.28 ± 0.23 across these BMI categories (*p* < 0.001), whereas FSH did not differ significantly (*p* = 0.07). BMI showed strong inverse correlations with LH (r = −0.870, *p* < 0.001) and the LH/FSH ratio (r = −0.869, *p* < 0.001), but not with FSH (r = −0.126, *p* = 0.075). Conclusions: Women with PCOS demonstrated a distinct gonadotropin profile characterized by higher LH and LH/FSH ratio and lower FSH compared with healthy controls. Among women with PCOS, increasing BMI was strongly associated with lower LH concentrations and LH/FSH ratios. These findings highlight the importance of considering BMI when interpreting gonadotropin profiles in PCOS, although prospective studies are required to determine whether changes in body weight directly influence gonadotropin secretion and reproductive outcomes.

## 1. Introduction

A high proportion of females at reproductive age globally suffer from the diverse endocrine condition known as polycystic ovarian syndrome (PCOS) [1]. The condition is frequently linked to high androgen levels, insulin resistance (IR), oversized and malfunctioning ovaries, etc. [2]. Around ten percent of women experience PCOS before to menopause and deal with its implications [3]. The Middle East and North Africa (MENA) region’s age-standardized point incidence and prevalence rates for PCOS reached 77.2 and 2079.7 per 100,000, accordingly, in 2019. These figures indicate growth of 33.7% and 37.9% since 1990 [4].

The significant economic cost associated with PCOS is highlighted by its elevated frequency and correlation with ovulation and menstruation irregularities, infertility, hair loss, and metabolic disorders [5]. The majority of instances of PCOS are identified between the ages of 20 and 30, however it can develop at any age, starting with menarche [6].

In small, young women without metabolic syndrome, PCOS can manifest as a separate condition. Nevertheless, it is often associated with metabolic problems, including obesity and weight gain, dysregulated glucose tolerance, IR, and disruption of the lipid profile. According to earlier research, nearly fifty percent of PCOS patients are obese [7,8]. Research from the United States shows that 35% of women in general are obese, compared to almost sixty percent of women with PCOS; additionally, 87.5% had a body mass index (BMI) of ≥26 kg/m^2^, which is almost twice as high as the entire population [9,10]. The average BMI of East Asian women with PCOS fluctuates between 20 and 22 kg/m^2^, which is significantly lower than the stated averages of 27.5 to 31.5 kg/m^2^ for Caucasians, reflecting the gap in the incidence of obesity [11,12]. Latest research has shown that obesity is becoming more common among women in the Gulf Cooperation Council (GCC) region, especially as a result of variables like dietary changes, inactivity, and urbanization [13]. According to the investigation on the prevalence of PCOS in MENA area during 1990 to 2019, females in the GCC countries are therefore probably more likely to develop the condition [4].

The National Institute of Health (NIH) and the World Health Organization (WHO) categorized obesity as having a BMI of thirty or higher, underweight as having a BMI of less than 18.5, normal weight as having a BMI among 18.5 and 24.9, and overweight as having a BMI between 25 and 29.9. Class 1 (BMI 30–34.9), Class 2 (BMI 35–39.9), and Class 3 (BMI > 40) are additional classifications for obesity [14].

However, PCOS individuals have far greater amounts of luteinizing hormone (LH) and the ratio of LH to follicle-stimulating hormone (FSH) [15,16]. This suggests that in lean PCOS individuals, neuroendocrine disorders could be the most significant factor. It has been documented that intrauterine hyperandrogen intake plays a major role in the reprogramming of several genes that contribute to the occurrence of PCOS, which impacts the hypothalamus-pituitary axis and metabolic disruption, triggering production of LH and the accumulation of abdominal fat and raising the danger of IR during adolescence and puberty [17]. Gonadotropin releasing hormone (GnRH) pulses generated by GnRH neurons in the hypothalamus influence anterior pituitary LH release. Additionally, women with PCOS have lower FSH, which raises the LH/FSH ratio, increases androgen synthesis from theca cells in the ovarium, and ultimately results in excessive androgen generation [18].

Nevertheless, additional research has shown an ongoing inverse connection between LH and obesity across a broad range of body weights in females with PCOS, confirming the theory that all oligomenorrheic PCOS patients have an intrinsic neuroendocrine dysfunction that is altered by weight gain [19,20,21]. Additionally, irregular menstruation, excess weight, and high blood LH levels are signs of PCOS [22]. It has long been known that PCOS is characterized by an elevation in LH relative to FSH secretion. A single test is unable to identify an elevated LH/FSH ratio due to the pulsatile nature of their secretion [19]. Taken together, the available evidence suggests that adiposity may modify the gonadotropin profile of women with PCOS; however, the extent to which BMI is associated with LH secretion and the LH/FSH ratio across different BMI levels remains incompletely characterized, particularly among women in Jordan. Therefore, the present study aimed to investigate the association between BMI and gonadotropin secretion, including serum LH, FSH, and the LH/FSH ratio, among women with PCOS and to compare these parameters with those of healthy women. Based on previous evidence regarding the effects of adiposity on reproductive endocrine function, the prespecified hypotheses were that: (1) women with PCOS would have a higher BMI, higher serum LH concentration, lower FSH concentration, and higher LH/FSH ratio than healthy controls; (2) among women with PCOS, increasing BMI would be inversely associated with serum LH concentration and the LH/FSH ratio; and (3) the association between BMI and FSH concentration would be weaker and would not reach statistical significance. These hypotheses were evaluated using comparisons between women with PCOS and healthy controls, comparisons across BMI categories within the PCOS group, and correlation analyses between BMI and gonadotropin parameters.

## 2. Patients and Methods

### 2.1. Study Design and Setting

In order to assess the relationship between BMI and gonadotropin measures, such as LH, FSH, and the LH/FSH ratio, among PCOS females in contrast to healthy controls, this research was carried out as a comparative cross-sectional analytical study. From June 2025 to April 2026, the research project was conducted in the outpatient gynecological and endocrinology clinics at medical centers in private sector in Irbid city in Jordan. Each step was carried out in compliance with accepted laboratory and clinical guidelines. All procedures were performed in accordance with the principles of the Declaration of Helsinki.

### 2.2. Study Population

The investigation comprised 400 women who were of reproductive age and were split into two primary groups. 200 women with PCOS diagnoses formed the patients’ group, and 200 similarly aged and healthy women made up the control population. Each groups’ members ranged in age from 18 to 35. BMI categories of <25, 25–<28, 28–<31, and ≥31 kg/m^2^ were used to subsequently stratify women with PCOS into four equivalent subgroups, each consisting of fifty individuals. Healthy females with normal menstruation, without biochemical or clinical signs of excessive estrogen production, and adequate ovarian morphology on USG made comprised the control population.

**Figure 1:**
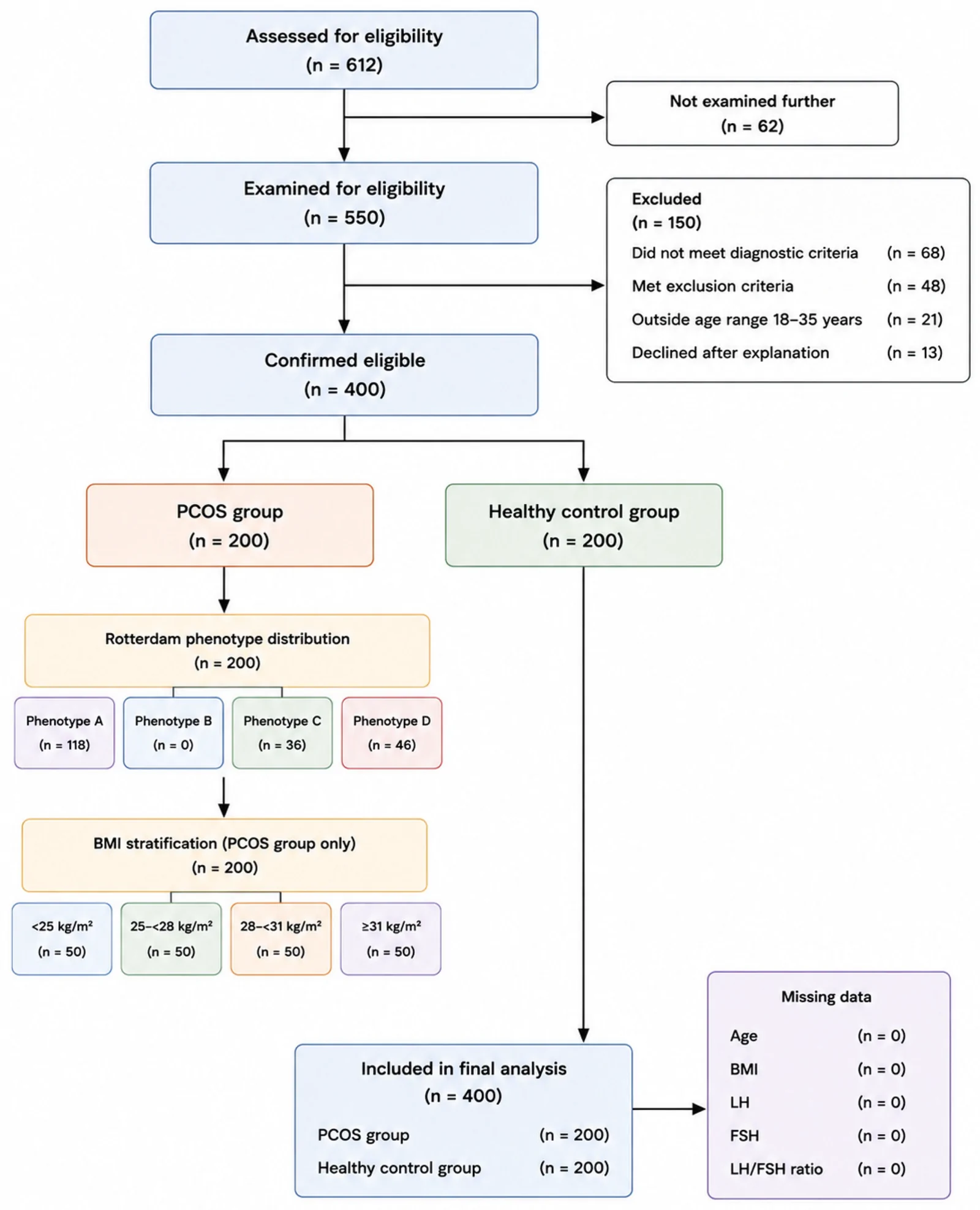
Flow diagram of the study participants.

The minimum required sample size was calculated using Epi Info™ version 7 (Centers for Disease Control and Prevention, Atlanta, GA, USA), using the Population Survey/Descriptive Study module. Assuming an expected frequency of 50%, a 95% confidence level, a 5% acceptable margin of error, a design effect of 1.0, and one cluster, the calculated minimum sample size was 384 participants. To provide an adequate sample for the planned comparative analyses and to allow equal allocation between the study groups, the final sample size was rounded to 400 participants, comprising 200 women with PCOS and 200 healthy controls. The PCOS group was subsequently divided equally into four BMI categories, with 50 participants in each category (Figure 1).

### 2.3. Diagnostic Criteria for Polycystic Ovary Syndrome

The Rotterdam diagnostic standards were used to diagnose PCOS [23]; therefore, after ruling out alternative causes, needed the existence of at least two of the following three characteristics: Menstrual irregularities, biochemical or clinical indicators of hyperandrogenism, and polycystic ovarian morphology on pelvic USG. Each PCOS subject in the study met the diagnostic requirements and had no other endocrine conditions that could account for their clinical manifestation.

Because the study population was recruited from women with polycystic ovarian morphology documented by pelvic ultrasonography, all participants in the PCOS group demonstrated PCOM. Consequently, the Rotterdam phenotype characterized by ovulatory dysfunction and hyperandrogenism without PCOM (phenotype B) was not represented in the present sample. The study was therefore designed to evaluate the relationship between BMI and gonadotropin parameters within the enrolled PCOS population with documented PCOM rather than to represent the full spectrum of Rotterdam PCOS phenotypes.

### 2.4. Inclusion and Exclusion Criteria

The PCOS group was accessible to women between the ages of 18 and 35 who had been diagnosed with PCOS. The control population consisted of healthy women who had consistent menstrual cycles, a normal BMI, and no clinical signs or evidence of PCOS or related endocrine problems. Cushing’s syndrome, congenital adrenal hyperplasia, hyperprolactinemia, thyroid gland problems, lactation, pregnancy, the consumption of insulin-responsive drugs or hormonal treatments during the previous three months, or any long-term chronic condition that could impact metabolic or hormonal indicators were among the exclusion factors for both groups.

### 2.5. Clinical and Anthropometric Assessment

Every participant had a thorough clinical assessment. Age and pregnancy history were among the demographic information that was documented. Consistent techniques were used to collect anthropometric measurements. Height was recorded to the nearest 0.1 cm utilizing a stadiometer and body weight was assessed to the nearest 0.1 kg utilizing a calibrated digital scale. Weight in kilograms divided by height in meters squared (kg/m^2^) was used to compute BMI. Medical history and physical assessment were used to record PCOS-associated clinical symptoms, such as infertility, acne, hirsutism, and irregular menstruation. Patients in the PCOS sample had their PCOS duration documented.

### 2.6. Ultrasonographic Evaluation

Pelvic ultrasonography was performed by experienced radiologists using a high-resolution GE Voluson E8 ultrasound system (GE HealthCare, Zipf, Tiefenbach, Austria) equipped with a RIC5-9-D multifrequency transvaginal probe. Transvaginal ultrasonography was performed whenever clinically appropriate, whereas transabdominal ultrasonography was used when transvaginal examination was not feasible or acceptable. Both ovaries were systematically examined in longitudinal and transverse planes, and ovarian morphology was assessed for follicular distribution and ovarian structure. Polycystic ovarian morphology (PCOM) was recorded when the ovarian appearance was consistent with the predefined sonographic criteria used by the study center. The ultrasound assessment was performed as part of the diagnostic evaluation of PCOS and was interpreted together with the clinical and hormonal findings rather than being used as the sole criterion for diagnosis.

### 2.7. Hormonal Assessment

Venous blood samples were collected from all participants under standardized conditions. In women with regular menstrual cycles, blood samples were obtained during the early follicular phase, between days 2 and 5 of the menstrual cycle. In women with oligomenorrhea or amenorrhea, samples were collected at the time of presentation because a predictable follicular-phase interval could not be established. Blood samples were allowed to clot and were subsequently centrifuged, and the separated serum was used for hormonal analysis.

Serum LH and FSH concentrations were measured using the automated VIDAS^®^ immunoassay system (bioMérieux, Marcy-l’Étoile, France), based on enzyme-linked fluorescent assay (ELFA) technology. Serum LH was measured using the VIDAS^®^ LH assay (Ref. 30406; bioMérieux), while FSH was measured using the VIDAS^®^ FSH assay (Ref. 30407; bioMérieux). According to the manufacturer’s specifications, the assays were intended for quantitative determination of LH and FSH in human serum. The analytical measurement ranges were 0.1–100 mIU/mL for LH and 0.1–110 mIU/mL for FSH. The assays were performed according to the manufacturer’s instructions using the manufacturer’s calibrators and quality-control materials. The LH/FSH ratio was calculated for each participant by dividing the measured serum LH concentration by the corresponding FSH concentration.

### 2.8. Data Management and Statistical Analysis

The data were collected, coded, and analyzed with the help of the Statistical Package for the Social Sciences (SPSS version 26). The categorial and continuous variables were calculated by percentages (along with chi, square test) and mean standard deviations (by independent sample t, test), respectively. Analysis of variance (ANOVA) one, way test for continuous parameters was utilized to compare PCOS patients within different BMI groups. The relationship between BMI and hormonal markers like LH, FSH, and the LH/FSH ratio was determined using Pearson’s correlation test. Statistical significance was considered when the probability value was less than 0.05.

## 3. Results

### 3.1. Comparison of Demographic, Clinical, and Hormonal Characteristics Between Women with PCOS and Healthy Controls

Table 1 displays the two groups’ age comparability between women with PCOS and healthy controls (26.31 ± 5.36 vs. 26.47 ± 5.16 years; *p* = 0.23). Moreover, the age distributions within the <25 years (41.5% vs. 35.5%), 2530 years (31.5% vs. 39.5%), and >30 years (27% vs. 25%) categories were almost the same. On the other hand, BMI was significantly higher in PCOS patients than in healthy women (27.85 ± 3.47 vs. 22.81 ± 2.29 kg/m^2^; *p* < 0.001). Thus, PCOS patients were equally distributed across BMI categories (<25, 25–<28, 28–<31, and ≥31 kg/m^2^; 25% each), while all controls had a BMI < 25 kg/m^2^. The clinical manifestations characterizing PCOS were menstrual irregularity (87.5%), hirsutism (79.5%), acne (78.5%), infertility (70%), and polycystic ovarian morphology on ultrasound (100%). The average duration of PCOS was 5.60 ± 3.28 years (range: 111 years). Hormonal test showed significantly higher LH levels in PCOS patients than in healthy women (8.44 ± 2.61 vs. 6.53 ± 1.16 IU/L; *p* < 0.001), accompanied by significantly lower FSH levels (4.64 ± 0.98 vs. 7.96 ± 1.18 IU/L; *p* < 0.001). LH/FSH ratio was markedly elevated in the PCOS group (1.84 ± 0.50 vs. 0.83 ± 0.20; *p* < 0.001) (Table 1).

### 3.2. Comparison of Clinical Characteristics and Gonadotropin Profiles Across BMI Categories Among Women with PCOS

Table 2 reveals that the age distribution was not significantly different among the four BMI categories of women with PCOS. The mean ages were similar and ranged from 25.58 ± 5.47 to 27.16 ± 5.20 years (*p* = 0.48), and there was no significant variation in age group distribution.

The prevalence of menstrual irregularity appeared to be equally high in all BMI groups, varying from 84% to 94%, and no statistically significant difference was found (*p* = 0.44). On the contrary, the occurrence of hirsutism varied significantly with the BMI category (*p* = 0.02), reaching the highest percentage of women with BMI < 25 kg/m^2^ (88%) and the lowest percentage of those with BMI ≥ 31 kg/m^2^ (64%).

Acne prevalence was similar across different BMI categories, with being present in 72% to 86% of patients (*p* = 0.27). Infertility was a frequent occurrence in all the groups, with a higher percentage of women with a BMI 28 kg/m^2^ being affected (76% in both 28–<31 and ≥31 kg/m^2^ groups), albeit the difference not being statistically significant (*p* = 0.31). The average duration of PCOS was also similar in the different BMI categories, between 4.90 ± 3.08 and 6.18 ± 3.23 years (*p* = 0.26). LH concentration displayed a marked and continuous drop as BMI grew, going down from 11.58 ± 1.26 IU/L in women with a BMI < 25 kg/m^2^ to 5.40 ± 1.29 IU/L in those with a BMI ≥ 31 kg/m^2^ (*p* < 0.001). FSH levels remained quite constant amongst the different BMI groups, with no statistically significant difference being observed (*p* = 0.07). Hence, the LH/FSH ratio also followed a significant reverse trend with BMI, gradually dropping from 2.46 ± 0.18 in the group with the lowest BMI to 1.28 ± 0.23 in that with the highest BMI (*p* < 0.001).

### 3.3. Association Between BMI and Gonadotropin Parameters Among Women with PCOS

**Figure 2:**
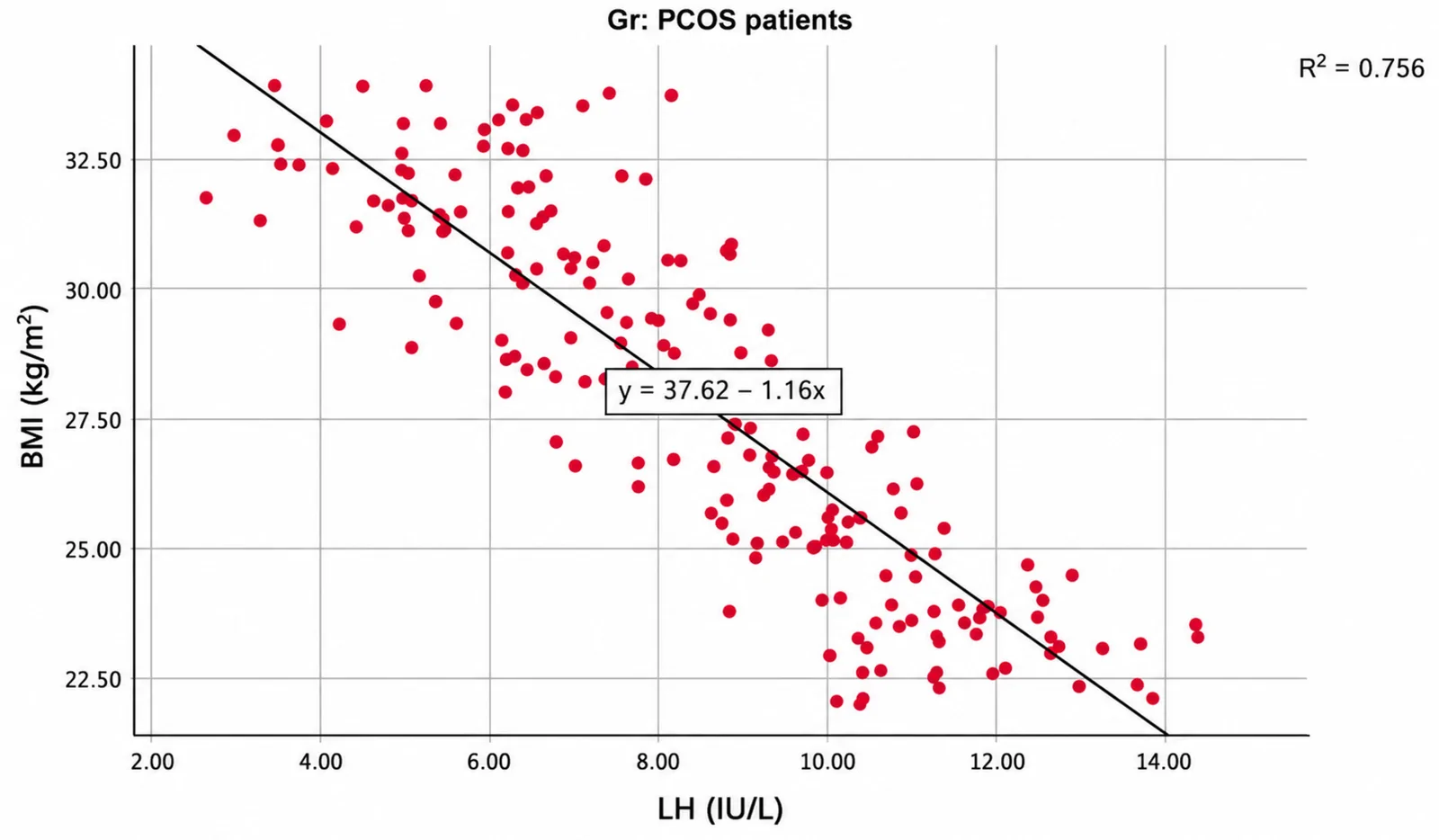
Scattered plot of the correlation between LH and BMI among PCOS patients.

**Figure 3:**
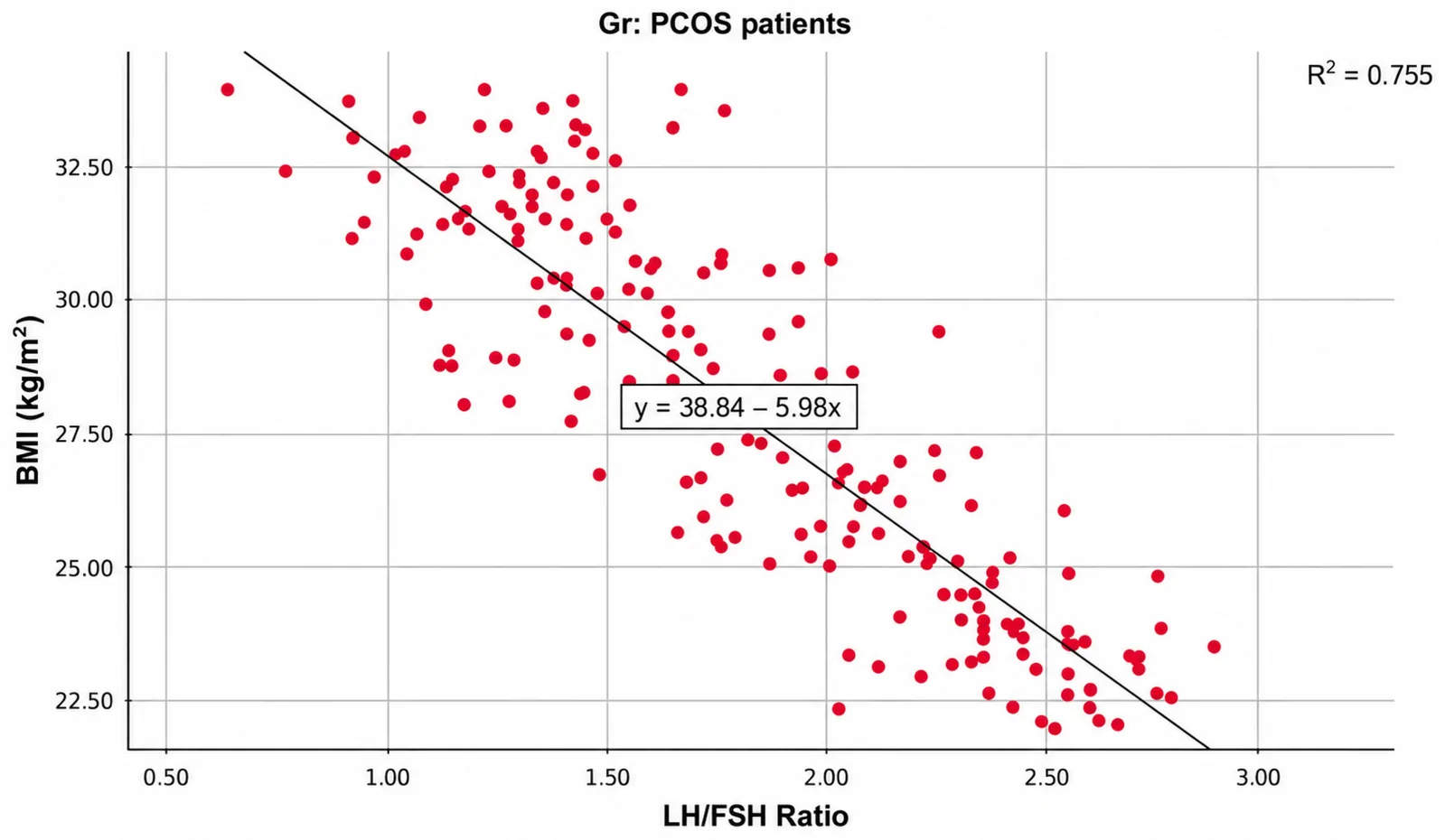
Scattered plot of the correlation between LH/FSH ratio and BMI among PCOS patients.

Table 3 and Figure 2 and Figure 3 demonstrated that BMI was strongly and inversely correlated with LH levels, showing a highly significant negative Pearson correlation coefficient (r = −0.870, *p* < 0.001). Similarly, BMI exhibited a strong and statistically significant inverse correlation with the LH/FSH ratio (r = −0.869, *p* < 0.001). In contrast, the correlation between BMI and FSH levels was weak and not statistically significant (r = −0.126, *p* = 0.075).

## 4. Discussion

Menstrual irregularities at the reproductive age are most frequently caused by PCOS, which is perhaps the most frequent endocrinological condition affecting women [24]. It is distinguished by having multiple ovaries on USG, as well as biochemical and clinical indications of oligo-anovulation and/or hyperandrogenism [25]. To our greatest knowledge, this is the first survey to measure blood LH levels, BMI, LH:FSH ratios, and the relationship between BMI and LH. FSH ratio in Jordanian patients with PCOS detected by USG.

In the current study, the mean age of PCOS patients was 26.31 ± 5.36 years with most patients (41.5%) aged <25 years.

Comparable findings were obtained by Parvin et al. (2022), who showed that the prevalent age range is 20–30 years old (77.6%) [21], which is the standard age of disease presence according to previous literature [4,22,26].

In the current study, hormonal test showed significantly elevated LH concentrations in PCOS patients than in healthy women, accompanied by significantly lower FSH levels. The LH/FSH ratio was markedly elevated in the PCOS group, ranging from 0.65 to 2.88, compared with controls.

These findings are supported by earlier research showing that the LH/FSH ratio is regarded as normal up to 1 [27,28,29]. Parvin et al. (2022) [21] found that the LH/FSH ratio was 2 in 46.6% of instances. Of the women with PCOS, 56.9% exhibited elevated concentrations of LH, whereas 43.1% maintained normal levels [21]. According to documented literature, females with PCOS has an increased mean serum level of LH [27,28,30,31,32].

In the current study, BMI was significantly higher among PCOS patients compared with healthy women. According to an investigation by Parvin et al. (2022), 75.9% of sufferers had BMIs between 25 and 28 kg/m^2^ BSA, whereas only 8.6% of patients had BMIs between 31 and 34 kg/m^2^ BSA [21]. This result is in line with several research conducted overseas wherein obesity is a prevalent observation [33,34,35,36].

In the current study, LH levels showed a significant progressive decline with increasing BMI. FSH levels were relatively similar across BMI categories, with no statistically significant difference observed. Consequently, the LH/FSH ratio also demonstrated a significant inverse association with BMI. As a result, BMI was significantly negatively correlated with LH levels, with a very highly significant negative Pearson correlation coefficient being observed. In the same way, BMI showed a strong and statistically significant inverse correlation with the LH/FSH ratio, thus suggesting a substantial decrease in LH predominance as body mass index increased.

According to Balen et al. (2003), PCOS women have disruptions in the standard gonadotrophin axis, which causes the amount of LH to rise and FSH levels to fall, reversing the LH/FSH ratio [37].

The findings supported the research by Lal et al. (2017) [38], which discovered significant differences in LH, FSH, and the LH/FSH ratio between obese and non-obese PCOS females. They recommended obese women who participated in the trial to strive to reduce their weight and alter their lifestyle in light of these outcomes [38].

Kiddy et al. discovered that hirsutism was more common in obese than normal or underweight women with PCOS and that there was a negative association between BMI and FSH concentrations among obese women [39].

In a nationwide study of postmenopausal females in the United States, it was discovered that the LH/FSH ratio was connected with PCOS-associated comorbidities such as dyslipidemia, IR, and obesity [40]. An ultrasound screening of 175 PCOS patients in Iran revealed that obese PCOS individuals were more likely than non-obese women to have a sonographic view of their polycystic ovaries [41].

Yuan et al. (2016) discovered that PCOS females who have a high BMI exhibit excessive androgens, which is consistent with the current findings [42]. Recent research has revealed a connection between PCOS and thyroid functioning [43,44].

In agreement with the present investigation, Parvin et al. (2022) discovered that the LH/FSH ratio declines with rising adiposity (∑ = −0.21, *p* < 0.009), suggesting a negative effect of BMI, and that increased LH cannot be regularly disclosed in PCOS women with BMI more than 30 kg/m^2^ [21].

Olooto et al. (2012) observed a significant variance between infertile and fertile females in LH (*p* = 0.00) and FSH (*p* = 0.00), but not in BMI (*p* = 0.554) [45]. This finding partially supported the present investigation [45].

The findings of Mahmoud et al. (2015), who found a statistically significant variance in BMI (*p* = 0.006) yet no difference in FSH and LH, are somewhat consistent with the present research [46].

In contrast to the present research, Mobeen et al. (2016) discovered that BMI was linked to obesity and hirsutism, whereas FSH was substantially correlated with infertility and obesity [19].

Saadia (2020) showed no change regarding the LH/FSH ratio (2.76 in normal BMI group opposite to 2.79 in elevated BMI patients, *p* = 0.48) or TSH, which contrasted with the current study [28].

According to a 2007 investigation conducted by Alnakash et al., there is no statistically significant relationship between BMI and the LH/FSH ratio [47], which disagreed with the present findings.

Begum et al. (2012) reported no variations in LH, FSH, or BMI (*p* > 0.05) [48], which contradicted with the current outcomes.

In contrast to the present examination, Alnakash’s (2007) research found no statistically significant differences in FSH, LH, or BMI among PCOS females [47].

Differences in sample sizes, laboratory techniques, and dietary and genetic factors may explain the differences in outcomes among studies. This accordingly leads to the urgent need for additional studies with large sample sizes to give support to the current findings.

The findings of the present study may have several implications for the clinical management of women with PCOS. In routine clinical practice, BMI assessment may be considered an important component of the initial evaluation and follow-up of women with PCOS, particularly when interpreting gonadotropin profiles. The observed progressive decline in LH concentration and LH/FSH ratio across increasing BMI categories suggests that body weight may modify the gonadotropin pattern in PCOS and should therefore be considered when interpreting LH and FSH results. In addition, women with PCOS and increased BMI may benefit from early counseling regarding healthy weight management, dietary modification, and regular physical activity as part of comprehensive PCOS care. Monitoring BMI together with menstrual regularity, reproductive symptoms, and relevant hormonal and metabolic parameters may help clinicians assess changes in the clinical phenotype over time. The findings may also support individualized counseling for women seeking fertility treatment, in whom obesity and reproductive endocrine abnormalities frequently coexist. However, because the present study was cross-sectional, the observed associations do not establish that weight reduction directly causes normalization of LH or the LH/FSH ratio. Therefore, prospective interventional studies are needed to determine whether intentional weight reduction produces clinically meaningful improvements in gonadotropin secretion and reproductive outcomes.

There are numerous benefits to the study. First of all, this is the first study in Jordan to detect the association between reproductive hormones (LH, FSH, and LH/FSH ratio) and PCOS patients’ obesity. Furthermore, adding a control group strengthens the findings and increases their accuracy and dependability. Moreover, a better understanding of the relationship between hormone levels and obesity was provided by splitting the group of PCOS patients into four subgroups based on BMI. Another advantage is the high, sufficient sample size, which reinforces the accuracy and generalizability of results. Additionally, by linking the reproductive hormones with BMI, clinicians are capable of diagnosing hypothalamic-pituitary ovarian dysfunction and early identify and treat patients with PCOS.

### Study Limitations

Several limitations of the present study should be acknowledged. First, the cross-sectional design limits the ability to establish temporal or causal relationships between BMI and gonadotropin alterations. Although strong inverse associations were observed between BMI and LH and between BMI and the LH/FSH ratio, these findings should not be interpreted as demonstrating that increasing BMI directly causes changes in gonadotropin secretion. Longitudinal and interventional studies are therefore needed to determine whether changes in body weight are accompanied by corresponding changes in gonadotropin secretion and reproductive outcomes. Second, and importantly, biochemical androgen levels were not assessed in the present study. Although clinical manifestations of hyperandrogenism, including hirsutism and acne, were recorded, the absence of biochemical measurements such as total or free testosterone and other circulating androgens limited the ability to comprehensively characterize the androgen status and Rotterdam phenotype of individual participants. This limitation is particularly relevant because androgen excess is a central component of the pathophysiology and diagnostic characterization of PCOS. Third, all women in the PCOS group had documented polycystic ovarian morphology on ultrasonography; consequently, the Rotterdam phenotype characterized by ovulatory dysfunction and hyperandrogenism without polycystic ovarian morphology (phenotype B) was not represented in the study population. Therefore, the findings should not be extrapolated to all PCOS phenotypes and are more appropriately interpreted as applying to the studied PCOS population with documented polycystic ovarian morphology. Fourth, insulin resistance and other important metabolic parameters were not assessed, despite their potential contribution to the interaction between adiposity, hyperandrogenism, and reproductive endocrine dysfunction in PCOS. Fifth, the study was conducted at medical centers in a single city in Jordan, which may limit the generalizability of the findings to women with PCOS in other geographical and ethnic populations. Finally, gonadotropin concentrations were assessed at a single time point despite the pulsatile nature of LH secretion, which may introduce biological variability in the measured LH concentration and LH/FSH ratio. Future multicenter, longitudinal studies incorporating comprehensive biochemical androgen assessment, metabolic profiling, repeated hormonal measurements, and representation of the full spectrum of Rotterdam phenotypes are warranted to further clarify the relationship between BMI and gonadotropin secretion in PCOS.

## 5. Conclusions

Women with PCOS had significantly higher BMI, LH concentrations, and LH/FSH ratios and lower FSH concentrations than healthy controls. Among women with PCOS, increasing BMI was strongly and inversely associated with LH concentration and the LH/FSH ratio, whereas the association between BMI and FSH was weak and not statistically significant. These findings suggest that BMI may be associated with variation in the gonadotropin profile among women with PCOS and documented polycystic ovarian morphology. However, the absence of biochemical androgen measurements and the lack of representation of the non-PCOM Rotterdam phenotype limit comprehensive phenotypic characterization and the generalizability of the findings to all women with PCOS. Further multicenter, longitudinal studies incorporating comprehensive androgen and metabolic assessment and including all Rotterdam phenotypes are warranted.

## 6. Recommendations

- To restore normal levels of LH, FSH, and LH/FSH ratios in women with PCOS, lifestyle modifications and weight control are recommended.
- Medical practitioners should reconsider how to return PCOS patients’ LH/FSH ratios to normal.
- It is necessary to conduct extensive, multicenter, and long, term studies to clarify the influence of BMI changes on hormonal anomalies related to reproduction in PCOS patients.
- In order to gain more insight into the link between metabolic disorders and reproductive hormones, it is necessary to investigate IR and other metabolic markers.

## Figures and Tables

**Table 1:** Demographic, Clinical, and Hormonal Characteristics of Women with PCOS and Healthy Controls.

Variable	Parameter	PCOS Patients N = 200	Healthy Women N = 200	*p*-Value (Test)
Age	Mean ± SD	26.31 ± 5.36	26.47 ± 5.16	0.23 (2.89)
	Min–max	18–35	18–35	
	<25 years	83 (41.5%)	71 (35.5%)	
	25–30 years	63 (31.5%)	79 (39.5%)	
	>30 years	54 (27%)	50 (25%)	
BMI (kg/m^2^)	Mean ± SD	27.85 ± 3.47	22.81 ± 2.29	<0.001 (293.52)
	Min–max	22.02–33.89	19.01–24.94	
	<25	50 (25%)	200 (100%)	
	25–<28	50 (25%)	0 (0%)	
	28–<31	50 (25%)	0 (0%)	
	≥31	50 (25%)	0 (0%)	
Menstrual Irregularity	Yes	175 (87.5%)	0 (0%)	-
	No	25 (12.5%)	200 (100%)	
Hirsutism	Present	159 (79.5%)	0 (0%)	-
	Absent	41 (20.5%)	200 (100%)	
Acne	Present	157 (78.5%)	0 (0%)	-
	Absent	43 (21.5%)	200 (100%)	
Infertility	Yes	140 (70%)	0 (0%)	-
	No	60 (30%)	200 (100%)	
Polycystic ovarian morphology on ultrasonography (PCOM)	Present	200 (100%)	0 (0%)	-
	Absent	0 (0%)	200 (100%)	
Duration of PCOS (years)	Mean ± SD	5.60 ± 3.28	-	-
	Min–max	1–11	-	
LH (IU/L)	Mean ± SD	8.44 ± 2.61	6.53 ± 1.16	<0.001 (90.29)
	Min–max	2.65–14.39	3.99–9.24	
FSH (IU/L)	Mean ± SD	4.64 ± 0.98	7.96 ± 1.18	<0.001 (925.22)
	Min–max	2.07–8.37	5.10–11.42	
LH/FSH ratio	Mean ± SD	1.84 ± 0.50	0.83 ± 0.20	<0.001 (677.32)
	Min–max	0.65–2.88	0.43–1.58	

The standard deviations and means represented the current data. Independent sample *t*-test was additionally performed to compare between continuous variables. Categorial data was presented as counts and percentages with χ^2^ for comparison.

**Table 2:** Clinical Characteristics and Gonadotropin Profiles of PCOS Patients Stratified by Body Mass Index Categories.

Variable	Parameter	<25 kg/m^2^ N = 50	25–<28 kg/m^2^ N = 50	28–<31 kg/m^2^ N = 50	≥31 kg/m^2^ N = 50	*p*-Value (Test)
Age (years)	Mean ± SD	27.16 ± 5.20	25.96 ± 5.14	26.54 ± 5.26	25.58 ± 5.47	0.48 (0.83)
	<25 years	16 (32%)	22 (44%)	21 (42%)	24 (48%)	
	25–30 years	19 (38%)	14 (28%)	15 (30%)	15 (30%)	
	>30 years	15 (30%)	14 (28%)	14 (28%)	11 (22%)	
Menstrual Irregularity	Yes	43 (86%)	43 (86%)	47 (94%)	42 (84%)	0.44 (2.69)
	No	7 (14%)	7 (14%)	3 (6%)	8 (16%)	
Hirsutism	Present	44 (88%)	41 (82%)	42 (84%)	32 (64%)	0.02 (10.40)
	Absent	6 (12%)	9 (18%)	8 (16%)	18 (36%)	
Acne	Present	43 (86%)	36 (72%)	41 (82%)	37 (74%)	0.27 (3.88)
	Absent	7 (14%)	14 (28%)	9 (18%)	13 (26%)	
Infertility	Yes	31 (62%)	33 (66%)	38 (76%)	38 (76%)	0.31 (3.62)
	No	19 (38%)	17 (34%)	12 (24%)	12 (24%)	
Duration of PCOS (years)	Mean ± SD	5.52 ± 3.51	6.18 ± 3.23	5.78 ± 3.25	4.90 ± 3.08	0.26 (1.34)
LH (IU/L)	Mean ± SD	11.58 ± 1.26	9.49 ± 0.98	7.30 ± 1.22	5.40 ± 1.29	<0.001 (249.44)
FSH (IU/L)	Mean ± SD	4.72 ± 0.63	4.78 ± 0.67	4.73 ± 1.22	4.32 ± 0.98	0.07 (2.37)
LH/FSH ratio	Mean ± SD	2.46 ± 0.18	2.01 ± 0.23	1.59 ± 0.27	1.28 ± 0.23	<0.001 (242.76)

The standard deviations and means represented the current data. Independent sample *t*-test was additionally performed to compare between continuous variables. Categorial data was presented as counts and percentages with χ^2^ for comparison.

**Table 3:** Correlation between Body Mass Index and Gonadotropin Parameters among Women with PCOS.

Variable	Parameter	BMI
LH	Pearson test	−0.870 **
	Value of significance	0.000
FSH	Pearson test	−0.126
	Value of significance	0.075
LH/FSH Ratio	Pearson test	−0.869 **
	Value of significance	0.000

Correlations were assessed using Pearson’s correlation coefficient with two-tailed significance testing. Statistical significance was set at *p* < 0.05. ** Correlation is significant at the 0.01 level (two-tailed).

## Data Availability

The data will be available for review from the corresponding author on request.

## References

[1] Deans R. (2019). Polycystic ovary syndrome in adolescence. Med. Sci..

[2] Witchel S.F., Oberfield S.E., Peña A.S. (2019). Polycystic ovary syndrome: Pathophysiology, presentation, and treatment with emphasis on adolescent girls. J. Endocr. Soc..

[3] Sadeghi H.M., Adeli I., Calina D., Docea A.O., Mousavi T., Daniali M., Nikfar S., Tsatsakis A., Abdollahi M. (2022). Polycystic ovary syndrome: A comprehensive review of pathogenesis, management, and drug repurposing. Int. J. Mol. Sci..

[4] Motlagh Asghari K., Nejadghaderi S.A., Alizadeh M., Sanaie S., Sullman M.J., Kolahi A.-A., Avery J., Safiri S. (2022). Burden of polycystic ovary syndrome in the Middle East and North Africa region, 1990–2019. Sci. Rep..

[5] Riestenberg C., Jagasia A., Markovic D., Buyalos R.P., Azziz R. (2022). Health care-related economic burden of polycystic ovary syndrome in the United States: Pregnancy-related and long-term health consequences. J. Clin. Endocrinol. Metab..

[6] Shukla A., Ashraf G.M., Sudharsan V., Arora T., Rather K.U.I., Chowdhury S., Suri V., Joshi B., Bhattacharya P.K., Agrawal S. (2025). Trends of Age at Onset of Menarche Among Indian Women of Reproductive Age and Its Association with the Presence of PCOS and Related Features: A Multicentric Cross Sectional Study: A. Shukla et al. J. Obstet. Gynecol. India.

[7] Glueck C.J., Goldenberg N. (2019). Characteristics of obesity in polycystic ovary syndrome: Etiology, treatment, and genetics. Metabolism.

[8] Smigoc K., Kawwass J.F. (2025). Polycystic ovarian syndrome, obesity, and insulin resistance: Intertwined comorbidities that impact assisted reproductive technology success. Fertil. Steril..

[9] Flegal K.M., Carroll M.D., Ogden C.L., Curtin L.R. (2010). Prevalence and trends in obesity among US adults, 1999–2008. JAMA.

[10] Azziz R., Sanchez L., Knochenhauer E., Moran C., Lazenby J., Stephens K., Taylor K., Boots L. (2004). Androgen excess in women: Experience with over 1000 consecutive patients. J. Clin. Endocrinol. Metab..

[11] Kim J.J., Choi Y.M. (2019). Phenotype and genotype of polycystic ovary syndrome in Asia: Ethnic differences. J. Obstet. Gynaecol. Res..

[12] Mani H., Davies M.J., Bodicoat D.H., Levy M.J., Gray L.J., Howlett T.A., Khunti K. (2015). Clinical characteristics of polycystic ovary syndrome: Investigating differences in White and South Asian women. Clin. Endocrinol..

[13] Mokhtar N., Elati J., Chabir R., Bour A., Elkari K., Schlossman N.P., Caballero B., Aguenaou H. (2001). Diet culture and obesity in northern Africa. J. Nutr..

[14] WHO Consultation on Obesity (2000). Obesity: Preventing and Managing the Global Epidemic: Report of a WHO Consultation.

[15] Moran C., Arriaga M., Arechavaleta-Velasco F., Moran S. (2015). Adrenal androgen excess and body mass index in polycystic ovary syndrome. J. Clin. Endocrinol. Metab..

[16] Tock L., Carneiro G., Pereira A.Z., Tufik S., Zanella M.T. (2014). Adrenocortical production is associated with higher levels of luteinizing hormone in nonobese women with polycystic ovary syndrome. Int. J. Endocrinol..

[17] Abruzzese G.A., Ferreira S.R., Ferrer M.J., Silva A.F., Motta A.B. (2023). Prenatal androgen excess induces multigenerational effects on female and male descendants. Clin. Med. Insights Endocrinol. Diabetes.

[18] Pratama G., Wiweko B., Asmarinah, Widyahening I.S., Andraini T., Bayuaji H., Hestiantoro A. (2024). Mechanism of elevated LH/FSH ratio in lean PCOS revisited: A path analysis. Sci. Rep..

[19] Mobeen H., Hamid H., Shafiq A., Adil M., Kashif M., Yousuf N.W. (2016). LH/FSH, BMI and clinical profile in polycystic ovarian syndrome: A correlative study. Int. J. Biochem. Res. Rev..

[20] Bohlke K., Cramer D.W., Barbieri R.L. (1998). Relation of luteinizing hormone levels to body mass index in premenopausal women. Fertil. Steril..

[21] Parvin R., Ahmmad B.N., Ara R., Begum M., Khatun R., Rashid M. (2022). Correlation between BMI and LH level and LH/FSH ratio-a cross-sectional study. TAJ J. Teach. Assoc..

[22] The Rotterdam ESHRE/ASRM-Sponsored PCOS Consensus Workshop Group (2004). Revised 2003 consensus on diagnostic criteria and long-term health risks related to polycystic ovary syndrome. Fertil. Steril..

[23] Smet M.E., McLennan A. (2018). Rotterdam criteria, the end. Australas. J. Ultrasound Med..

[24] Stener-Victorin E., Teede H., Norman R.J., Legro R., Goodarzi M.O., Dokras A., Laven J., Hoeger K., Piltonen T.T. (2024). Polycystic ovary syndrome. Nat. Rev. Dis. Primers.

[25] Bai H., Ding H., Wang M. (2024). Polycystic ovary syndrome (PCOS): Symptoms, causes, and treatment. Clin. Exp. Obstet. Gynecol..

[26] Sanad A.S. (2014). Prevalence of polycystic ovary syndrome among fertile and infertile women in Minia Governorate, Egypt. Int. J. Gynecol. Obstet..

[27] Malini N., George K.R. (2018). Evaluation of different ranges of LH: FSH ratios in polycystic ovarian syndrome (PCOS)–Clinical based case control study. Gen. Comp. Endocrinol..

[28] Saadia Z. (2020). Follicle stimulating hormone (LH: FSH) ratio in polycystic ovary syndrome (PCOS)-obese vs. non-obese women. Med. Arch..

[29] Dunaif A., Thomas A. (2001). Current concepts in the polycystic ovary syndrome. Annu. Rev. Med..

[30] Yen S., Vela P., Rankin J. (1970). Inappropriate secretion of follicle-stimulating hormone and luteinizing hormone in polycystic ovarian disease. J. Clin. Endocrinol. Metab..

[31] Mohammed Z.I., Qasim M.T. (2021). Correlation of AMH and LH levels in PCOS patients with pregnancy rate. Ann. Rom. Soc. Cell Biol..

[32] Yang J., Chen C. (2024). Hormonal changes in PCOS. J. Endocrinol..

[33] Kopelman P.G. (1994). 4 Hormones and obesity. Baillieres Clin. Endocrinol. Metab..

[34] Chen X., Ni R., Mo Y., Li L., Yang D. (2010). Appropriate BMI levels for PCOS patients in Southern China. Hum. Reprod..

[35] Neubronner S.A., Indran I.R., Chan Y.H., Thu A.W.P., Yong E.-L. (2021). Effect of body mass index (BMI) on phenotypic features of polycystic ovary syndrome (PCOS) in Singapore women: A prospective cross-sectional study. BMC Women’s Health.

[36] Durmus U., Duran C., Ecirli S. (2017). Visceral adiposity index levels in overweight and/or obese, and non-obese patients with polycystic ovary syndrome and its relationship with metabolic and inflammatory parameters. J. Endocrinol. Investig..

[37] Balen A.H., Laven J.S., Tan S.L., Dewailly D. (2003). Ultrasound assessment of the polycystic ovary: International consensus definitions. Hum. Reprod. Update.

[38] Lal L., Bharti A., Perween A. (2017). To study the status of LH: FSH ratio in obese and non-obese patients of polycystic ovarian syndrome. IOSR J. Dent. Med. Sci..

[39] Kiddy D., Sharp P., White D., Scanlon M., Mason H., Bray C., Polson D., Reed M., Franks S. (1990). Differences in clinical and endocrine features between obese and non-obese subjects with polycystic ovary syndrome: An analysis of 263 consecutive cases. Clin. Endocrinol..

[40] Beydoun H.A., Beydoun M.A., Wiggins N., Stadtmauer L. (2012). Relationship of obesity-related disturbances with LH/FSH ratio among post-menopausal women in the United States. Maturitas.

[41] Esmaeilzadeh S., Andarieh M.G., Ghadimi R., Delavar M.A. (2014). Body mass index and gonadotropin hormones (LH & FSH) associate with clinical symptoms among women with polycystic ovary syndrome. Glob. J. Health Sci..

[42] Yuan C., Liu X., Mao Y., Diao F., Cui Y., Liu J. (2016). Polycystic ovary syndrome patients with high BMI tend to have functional disorders of androgen excess: A prospective study. J. Biomed. Res..

[43] Cai J., Zhang Y., Wang Y., Li S., Wang L., Zheng J., Jiang Y., Dong Y., Zhou H., Hu Y. (2019). High thyroid stimulating hormone level is associated with hyperandrogenism in euthyroid polycystic ovary syndrome (PCOS) women, independent of age, BMI, and thyroid autoimmunity: A cross-sectional analysis. Front. Endocrinol..

[44] Yu Q., Wang J.-B. (2016). Subclinical hypothyroidism in PCOS: Impact on presentation, insulin resistance, and cardiovascular risk. BioMed Res. Int..

[45] Olooto W.E., Adeleye A.O., Amballi A.A., Mosuro A.O. (2012). Pattern of reproductive hormones (follicle stimulating hormone, luteinizing hormone, estradiol, progesterone, and prolactin) levels in infertile women in Sagamu South Western Nigeria. Der Pharm. Lett..

[46] Mahmoud M.I., Habeeb F., Kasim K. (2015). Reproductive and biochemical changes in obese and non obese polycystic ovary syndrome women. Alex. J. Med..

[47] Alnakash A., Al-Tae’e N.K. (2007). Polycystic ovarian syndrome: The correlation between the LH/FSH ratio and disease manifestations. Middle East Fertil. Soc. J..

[48] Begum S., Hossain M.Z., Rahman M.F., Banu L.A. (2012). Polycystic ovarian syndrome in women with acne. J. Pak. Assoc. Dermatol..

